# Ventilator-associated pneumonia in Polish intensive care unit dedicated to COVID-19 patients

**DOI:** 10.1186/s12890-023-02743-7

**Published:** 2023-11-16

**Authors:** Marta Wałaszek, Piotr Serwacki, Zbigniew Cholewa, Alicja Kosiarska, Wioletta Świątek – Kwapniewska, Małgorza Kołpa, Elżbieta Rafa, Róża Słowik, Karolina Nowak, Anna Różańska, Jadwiga Wójkowska-Mach

**Affiliations:** 1 University of Applied Sciences in Tarnów, ul. Mickiewicza 8, Tarnów, 33-100 Poland; 2 St Luke Regional Hospital in Tarnów, ul. Lwowska 178A, Tarnów, 33-100 Poland; 3 University of Applied Sciences in Nowy Sącz, ul. Staszica 1, Nowy Sącz, 33-300 Poland; 4 Department of Microbiology, Jagiellonian University Medical College, Czysta str. 18, Krakow, 31-121 Poland

**Keywords:** Healthcare-associated pneumonia, Mechanical ventilation, COVID-19, Infection control

## Abstract

**Background:**

Healthcare-Associated Infections (HAI) are most frequently associated with patients in the Intensive Care Unit (ICU). Coronavirus disease 2019 (COVID-19), caused by Severe Acute Respiratory Syndrome Coronavirus 2 (SARS-CoV-2), led to ICU hospitalization for some patients.

**Methods:**

The study was conducted in 2020 and 2021 at a hospital in southern Poland. The Healthcare-Associated Infections Surveillance Network (HAI-Net) of the European Centre for Disease Prevention and Control (ECDC) was used for HAI diagnosis. The aim of this case-control study was to retrospectively assess the epidemiology of HAIs in ICU patients, distinguishing between COVID-19 and non-COVID-19 cases.

**Results:**

The study included 416 ICU patients: 125 (30%) with COVID-19 and 291 (70%) without COVID-19, *p* < 0.05. The mortality rate was 80 (64%) for COVID-19 patients and 45 (16%) for non-COVID-19 patients, *p* < 0.001. Ventilator-Associated Pneumonia (VAP) occurred in 40 cases, with an incidence rate density of 6.3/1000 patient-days (pds): 14.1/1000 pds for COVID-19 patients vs. 3.6/1000 pds for non-COVID-19 patients. Odds Ratio (OR) was 2.297, *p* < 0.01. Acinetobacter baumannii was the most often isolated microorganism in VAP, with 25 cases (incidence rate 8.5%): 16 (18.2%) in COVID-19 patients vs. 9 (4.4%) in non-COVID-19 patients. OR was 4.814 (1.084–4.806), *p* < 0.001.

**Conclusions:**

Patients treated in the ICU for COVID-19 faced twice the risk of VAP compared to non-COVID-19 patients. The predominant microorganism in VAP cases was Acinetobacter baumannii.

## Background

Coronaviruses, members of the Coronaviridae family, have been recognized in contemporary medicine since the 1960s [1]. In the realm of infectious diseases, coronaviruses were previously responsible for approximately 20% of upper respiratory tract infections in both children and adults. However, a significant paradigm shift occurred at the beginning of 2019 when cases of acute, unexplained lung inflammation emerged in China. This novel threat was identified as a new type of coronavirus, Severe Acute Respiratory Syndrome Coronavirus 2 (SARS-CoV-2) [2], leading to the nomenclature of Coronavirus Disease 2019 (COVID-19) by the World Health Organization (WHO).

For some patients, SARS-CoV-2 infection manifested as Acute Respiratory Distress Syndrome (ARDS), necessitating treatment in Intensive Care Units (ICUs). Patients in ICUs are at risk of invasive procedures, including mechanical ventilation (MV), which may result in nosocomial pneumonia (NP), specifically Ventilator-Associated Pneumonia (VAP).

A study conducted by Guan et al. [3] in China at the early stages of the pandemic (January 2020) revealed that 5% of COVID-19 patients required ICU admission, with 2.3% undergoing mechanical ventilation. It is estimated that approximately 20% of patients experience a severe or very severe course of the disease, primarily characterized by gas exchange disorders, notably hypoxemia [4].

The experience gained from hospitals during the COVID-19 pandemic underscores the frequent occurrence of nosocomial pneumonias as Healthcare-Associated Infections (HAI), often associated with high mortality rates [5–8]. The incidence rate of NP in ICUs typically ranges from 8.0 to 10.0% [8, 9]. Surveillance data from European ICUs indicate VAP incidence rates between 1.3% and 6.3% in various European countries [10].

The aim of this case-control study is to retrospectively analyze the epidemiology of VAP in patients treated in 2020–2021, categorizing them into COVID-19 and non-COVID-19 groups.

## Methods

This analysis is based on the results of a two-year surveillance conducted in the ICU of St. Luke Regional Hospital in Tarnów in 2020 and 2021.

Patients diagnosed with COVID-19 were accommodated in the ICU in a dedicated nine-person room with specialized medical staff, sanitary, and hygienic facilities. Non-COVID-19 patients in the ICU were treated in a five-person room with their own specialized personnel and facilities. These two groups of patients and their respective medical personnel did not interact.

Active, continuous, and targeted surveillance of Healthcare-Associated Infections (HAI) was conducted. Approximately 50% of nurses in the ward treating COVID-19 patients were transferred from other non-ICU hospital wards. Data on patients and hospital infections were collected as part of an active and targeted surveillance process following the standardized protocol established by the European Centre for Disease Prevention and Control (ECDC), version 4.3 [11]. The definition of a hospital-acquired infection, as per the implementing decision of the European Commission in 2018, was applied [12]. Patients with an ICU stay of fewer than 48 h were excluded from the analysis.

The following types of HAIs were monitored: hospital-acquired pneumonia (Pneumonia NP), hospital-acquired bloodstream infection (Bloodstream Infections BSI), Urinary Tract Infections (UTI), Surgical Site Infections (SSI), Gastrointestinal Infections (GI), Skin and Soft Tissue Infections (SST), Lower Respiratory Tract Infections (LRI), and Systemic Infections (SYS).

### Statistical analysis

A retrospective statistical analysis was performed using IBM SPSS (SPSS – Statistical Package for the Social Sciences, STATISTICS 24, Armonk, NY, USA) and Microsoft Excel (Microsoft Office 2016 Redmond, WA, USA). Statistical calculations included frequencies (n), percentages (%), medians (Me), standard deviations (SD), significance levels (p), where *p* < 0.05 indicated statistical significance. The analysis involved calculating odds ratios (OR) and 95% confidence intervals (95% CI) for both groups, classified by the presence or absence of HAI. Fisher’s exact probability test was used due to sample size considerations.

Incidence rates were calculated for VAP, indicating the number of new cases per 100 admissions in the ICU, as well as incidence density rates, reflecting the number of new VAP cases per 1000 patient-days with mechanical ventilation. Additionally, utilization rates (UR) for patients with mechanical ventilation (MV) were calculated as the number of days with the procedure per 100. A minimum sample size of 399 hospitalized patients was required for this study.

The data used for analysis were anonymized. The study was based on routinely collected hospitalization data, obviating the need for additional consent for usage.

The study was conducted with the approval of the Bioethics Commission of the Jagiellonian University in Krakow (no KBET 1075.6120.12.2023) and adhered to the principles of the Helsinki Declaration [13].

## Results

From January 1, 2020, to December 31, 2021, a total of 416 patients who met the study criteria were admitted to the ICUs. Of these, 125 patients (30.0%) were diagnosed with COVID-19, while 291 patients (70.0%) were non-COVID-19 cases (*p* < 0.05). Notably, the patients admitted with SARS-CoV-2 were not vaccinated against COVID-19. COVID-19 patients were generally older (median age 68 years) compared to non-COVID-19 patients (median age 62), which was statistically significant (*p* < 0.001). Among patients with COVID-19, males were predominant. The duration of ICU stay was shorter for COVID-19 patients (median 17 days) compared to non-COVID-19 patients (median 22 days), with a statistically significant difference (*p* < 0.05). The death rate was significantly higher among COVID-19 patients (64.0%) compared to non-COVID-19 patients (15.5%) (*p* < 0.001). The incidence rate of HAI was comparable in both groups (32.8% in COVID-19 patients vs. 30.2% in non-COVID-19 patients; *p* = 0.332). Notably, broncho-alveolar lavage (BAL) tests were performed in a lower percentage of COVID-19 patients (5%) compared to non-COVID-19 patients (20%) (see Table 1).

**Table 1:** Demographic characteristic of ICU patients, their number, hospitalization patientdays, incidence rate per 100 cases of hospitalization, death rate in 2020–2021

Description of the ward				
	non-COVID-19 patient	COVID-19 patient		*P*
Number of patients and patientdays of hospitalization				
Number of patients in ICU in 2020 n (%)	145 (75.5)	47( 24.5)	192 (100.0)	< 0.05
Number of patients in ICU in 2021 n (%)	146 (65.2)	78 (34.8)	224 (100.0)	
Total number of patients in ICU in 2020–2021 n (%)	291 (70.0)	125 (30.0)	416 (100.0)	
Description of patients				
Age of patients: median (SD)	62 (19.4)	68 (12.9)	64 (18.1)	< 0.001
Gender W/M	0.5	0.7	0.5	0.172
Days of hospitalization: median (SD),	22 (24.5)	17 (25.3)	19 (24.8)	< 0.05
Number of deaths (death rate) n (%)	45 (15.5)	80 (64.0)	125 (100.0)	< 0.001
Healthcare associated infections				
Number of healthcare associated infections	88	41	129	0.332
Incidence rate per 100 patientdays	30.2	32.8	31.0	
Bronchoscopy				
Bronchoscopy n (%)	58 (19.9)	6 (4.8)	64 (15.4)	< 0.001

*SD* Standard deviation, *ICU* Intensive care unit, *W* Woman, *M* Man, *UR* Utilization rate, *HAI* Healthcare-Associated Infections

Various forms of HAI were identified, i.e. Pneumonia (PN) 40 (9.6%) cases including: 18 (14.4%) with COVID-19 vs. 22 (7.6%) non-COVID-19, *p* < 0.05, Bloodstream Infection (BSI) 37 (8.9%) cases, including: 10 (8.0%) with COVID-19 vs. 27 (9.3%) non-COVID-19, *p* = 0.791; Urinary Tract Infection (UTI) 26 (6.3%) cases, including: 7 (5.6%) with COVID-19 vs. 19 (6.5%) non-COVID-19, *p* = 0.806; Gastrointestinal system infection – *Clostridioides difficile* (GI-CDI) 8 (1.9%) cases, including: 2 (1.6%) with COVID-19 vs. 6 (2.1%) non-COVID-19, *p* = 0.578; Systemic infection (SYS) 9 (2.2%) cases, including: 1 (0.8%) with COVID-19 vs. 8 (2.7%) non-COVID-19, *p* = 0.294; Skin and Soft Tissue infection (SST) 6 (1.4%) cases, including: 1 (0.8%) with COVID-19 vs. 5 (1.7%) non-COVID-19, *p* = 0.677; Surgical Site Infection (SSI) 1 (0.2%) case, including 0 (0.0%) with COVID-19 vs. 1 (0.3%) non-COVID-19; Lower Respiratory Tract Infection (LRI) 2 (0.5%) cases, including: 2 (1.6%) with COVID-19 vs. 0 (0.0%) non-COVID-19.

Among the etiological factors, particularly noteworthy were non-fermenters, with *Acinetobacter baumannii* being the dominant pathogen, accounting for 47 (36.4%) of the cases (see Table 3).

Patients diagnosed with COVID-19 who required intensive care experienced a shorter duration of invasive mechanical ventilation compared to patients treated for other medical conditions. The utilization rates (UR) were notably lower in COVID-19 patients (0.36) compared to non-COVID-19 patients (0.94). The incidence rate of Ventilator-Associated Pneumonia (VAP) was significantly higher in COVID-19 patients, with an incidence rate of 14.1 per 1000 patient-days with a ventilator, in contrast to the lower rate of 3.6 per 1000 patient-days in non-COVID-19 patients (as detailed in Table 4). The basis for microbiological diagnosis of VAP in patients with COVID-19 was the material from lower airways, 18 (100%) (see Tables 3 and 4).

**Fig. 1:**
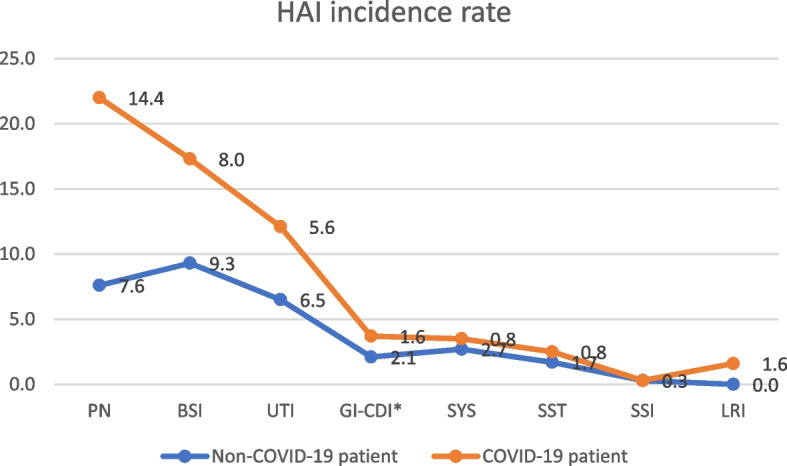
Healthcare-associated infections incidence rates in COVID-19 vs. Non-COVID-19 patients

**Fig. 2:**
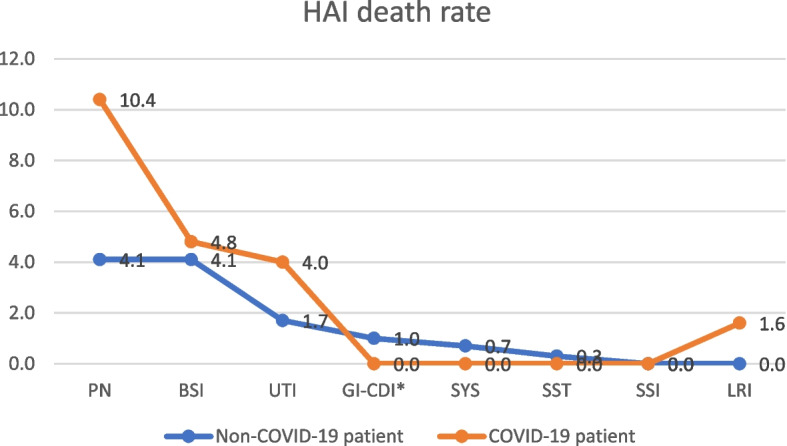
Healthcare-associated infections death rates in COVID-19 vs. Non-COVID-19 patients

The mortality rate among patients treated for COVID-19 with HAIs was nearly twofold higher compared to non-COVID-19 patients, OR = 2.624 (95% confidence interval (CI) 1.221–5.644, *p* < 0.05). The death rate for hospital-acquired PN was twofold higher, OR = 2.325 (95% CI 1.199–7.205), *p* < 0.05 (see Table 2, Figs. 1 and 2). The incidence rate of HAIs attributed to *Acinetobacter baumannii* was three times higher among COVID-19 patients compared to non-COVID-19 patients, OR = 3.342 (95% CI 1.799–6.208, *p* < 0.001, Table 3). The VAP incidence rate was found to be two times higher among COVID-19 patients than in the case of non-COVID-19 patients, reflected in an Odds Ratio (OR) of 2.297 (95% CI 1.236–4.267, *p* < 0.01, Table 4). Notably, the incidence rate of HAIs linked to *Acinetobacter baumannii* was four times higher among COVID-19 patients compared to non-COVID-19 patients, OR = 4.814 (95% CI 2.037–11.380, *p* < 0.001, Table 4).

**Table 2:** Clinical forms, number of HAI, HAI incidence rate per 100 cases of hospitalization and HAI death rate in ICU in 2020–2021

HAI type	non-COVID-19 patient	COVID-19 patient	Total	Odds Ratio (OR) 95% confidence interval	*P*
HAI incidence rate n (%)					
PN	22 (7.6)	18 (14.4)	40 (9.6)	2,103 (1,084 − 4,806)	< 0.05
BSI	27 (9.3)	10 (8.0)	37 (8.9)	0.903 (0.425–1.918)	0.791
UTI	19 (6.5)	7 (5.6)	26 (6.3)	0.899 (0.369–2.188)	0.806
GI-CDI^a^	6 (2.1)	2 (1.6)	8 (1.9)	0.819 (0.162–4.081)	0.578
SYS	8 (2.7)	1 (0.8)	9 (2.2)	0.271 (00.34–2.161)	0.294
SST	5 (1.7)	1 (0.8)	6 (1.4)	0.488 (0.056–4.217)	0.677
SSI	1 (0.3)	0 (0.0)	1 (0.2)	nd	nd
LRI	0 (0.0)	2 (1.6)	2 (0.5)	nd	nd
Total	88 (30.2)	41 (32.8)	129 (31.0)	1.273 (0.821–1.975)	0.332
Death rate n (%)					
PN	12 (4.1)	13 (10.4)	25 (6.0)	2.325 (1.199–7.205)	< 0.05
BSI	12 (4.1)	6 (4.8)	18 (4.3)	1.085 (0.376–3.129)	0.920
UTI	5 (1.7)	5 (4.0)	10 (2.4)	2.371 (0.645–8.710)	0.284
GI-CDI^a^	3 (1.0)	0 (0.0)	3 (0.7)	nd	nd
SYS	2 (0.7)	0 (0.0)	2 (0.5)	nd	nd
SST	1 (0.3)	0 (0.0)	1 (0.2)	nd	nd
SSI	0 (0.0)	0 (0.0)	0 (0.0)	nd	nd
LRI	0 (0.0)	2 (1.6)	2 (0.5)	nd	nd
Total	35 (12.0)	26 (20.8)	61 (14.7)	2.624 (1.221–5.644)	< 0.05

*HAI* Healthcare-Associated Infections, *PN* Pneumonia – lungs infection, *BSI* Bloodstream infection, *UTI* Urinary tract infection, *GI* Gastrointestinal system infection, *SYS* Systemic infection, *SST* Skin and soft tissue, *SSI* Surgical site infection, *LRI* Lower respiratory tract infection, *n* number, *SD* Standard deviation

^a^*Clostridioides difficile* Infection (GI-CDI)- 8 cases/ incidence rate per 10 000 patientdays = 8.3 (GI-CDI)

**Table 3:** Microorganism responsible for HAI in ICU in 2020–2021

Microorganism type	non-COVID-19 patient	COVID-19 patient	Total
	n(%)	n(%)	n(%)
**Gram-positive cocci n (%)**	32 (36.4)	8 (19.5)	40 (31.0)
* Staphylococcus aureus*	12 (13.6)	3 (7.3)	15 (11.6)
* Staphylococcus epidermidis*	10 (11.4)	1 (2.4)	11 (8.5)
Enterococcus faecalis	8 (9.1)	2 (4.9)	10 (7.8)
* Enterococcus faecium*	1 (1.1)	2 (4.9)	3 (2.3)
* Streptococcus pneumoniae*	1 (1.1)	0 (0.0)	1 (0.8)
**Enterobacteriaceae n (%)**	18 (20.5)	2 (4.9)	20 (15.5)
* Klebsiella pneumoniae*	8 (9.1)	1 (2.4)	9 (7.0)
* Klebsiella aerogenes*	1 (1.1)	0 (0.0)	1 (0.8)
* Escherichia coli*	6 (6.8)	0 (0.0)	6 (4.7)
* Enterobacter cloacae*	3 (3.4)	1 (2.4)	4 (3.1)
**Gram-negative glucose non-fermenting bacteria n (%)**	24 (27.30	26 (63.4)	50 (38.8)
* Pseudomonas aeruginosa*	3 (3.4)	0 (0.0)	3 (2.3)
* Acinetobacter baumannii*^a^	21 (23.9)	26 (63.4)	47 (36.4)
**Other n (%)**	13 (14.8)	5 (12.2)	18 (14.0)
* Candida spp.*	7 (8.0)	3 (7.3)	10 (7.8)
* Clostidioides difficile*	6 (6.8)	2 (4.9)	8 (6.2)
**No microbiological confirmation n (%)**	1 (1.1)	0 (0.0)	1 (0.8)
NO GROWTH OF MICROORGANISM	1 (1.1)	0 (0.0)	1 (0.8)
**Total**	**88 (100.0)**	**41 (100.0)**	**129 (100.0)**
PN based on the microbiological diagnosis [11]			
PN1 - positive quantitative culture from minimally contaminated lower respiratory tract specimen such as broncho-alveolar lavage	5 (22.7)	0 (0.0)	5 (12.5)
PN2 - protected brush or distal protected aspirate, endotracheal aspirate (ETA) non-protected sample with quantitative culture	15 (68.2)	18 (100.0)	33 (82.5)
PN3 - alternative microbiological criteria	1 (4.5)	0 (0.0)	1 (2.5)
PN4 - sputum bacteriology or non-quantitative culture	0 (0.0)	1 (0.0)	0 (0.0)
PN5 – no results of microbiological tests	1 (4.5)	2 (0.0)	1 (2.5)
Total	22 (100.0)	18 (100.0)	40 (100.0)
Incidence rate of *Acinetobacter baumannii*^a^ n (%)			
2020	11 (7.6)	8 (17,1)	19 (9.9)
2021	10 (6.9)	18 (23.1)	28 (12.5)
**Total in 2022 − 2021 n (%)**	**21 (7.2)**	**26 (20.8)**	**47 (11.3)**
Odds Ratio (OR) for inception rate of *Acinetobacter baumannii/100hospitalization cases*	OR = 3.342 (1.799–6.208) *p* < 0.001		

^a^ Incidence rate of *A. baumannii* in patients in the investigated ICU in 2012–2019 was 4.2%

**Table 4:** Analysis of healthcare-associated infections related to the use of mechanical ventilation in ICU in 2020–2021

	non-COVID-19 patient	COVID-19 patient	Total	Odss Ratio (OR)
Number and percent of mechanically ventilated patients n(%)	204 (70.1)	88 (70.4)	292 (70.2)	0.132
VAP number	17	23	40	2.297 (1.236–4.267), *p* < 0.01
VAP incidence rate per 100 patients	8.3	26.1		
VAP incidence rate per 1000 days of MV (%)	3.6	14.1	6.3	
Persondays with mechanical ventilation (n)	4753	1633	6 386	nd
Number of persondays of hospitalization	5052	4543	9 595	nd
UR for mechanical ventilation (%)	0.94	0.36	0.67	nd
Number of VAP with *Acinetobacter baumannii* aetiology	9	16	25	4.814 (2.037–11.380), *p* < 0.001
*Acinetobacter baumannii* incidents rate in VAP / 100 patients n (%)	4.4	18.2	8.5	

*PN* Pneumonia, *VAP* Ventilator-associated pneumonia, *MV* Mechanical ventilation, *UR* Utilisation rate

## Discussion

From our study population, which consisted of patients admitted to the ICU with SARS-CoV-2 infection, none had been vaccinated against COVID-19. Vaccination against COVID-19 significantly lowers the risk of severe disease. Consequently, the studied COVID-19 patients were more exposed to a severe course of SARS-CoV-2 infection and the risk of being treated in the ICU. Poland’s COVID-19 vaccination coverage is relatively low compared to other countries. According to the European Centre for Disease Prevention and Control (ECDC), only 61% of the Polish population has received at least one dose [14]. In the city of Tarnów, where this study took place, vaccination coverage was 49%, while outside the city, it was no more than 41% [15]. These low vaccination rates are likely a consequence of the increasing influence of anti-vaccination movements. These movements have undermined public trust in vaccinations and led to a rise in refusals of mandatory immunizations [16, 17]. This situation has significantly burdened the Polish healthcare system and has become a significant public health issue.

The HAI incidence rate per 100 hospitalized patients in our study was 31%, and it was similar in both the group of COVID-19 patients (33%) and the group of non-COVID-19 patients (30%). Another Polish study conducted in two ICUs among COVID-19 patients reported a considerably higher HAI incidence rate at 56% [18]. Similarly, studies from other European countries have also shown high HAI incidence rates during the COVID-19 pandemic among patients hospitalized in the ICU [19, 20]. For instance, Grasselli et al. [19], in a multicenter study across 8 Italian hospitals, reported an ICU incidence rate of 46% among COVID-19 patients. In a single-center study in Spain, patients treated in the ICU due to COVID-19 had a 41% HAI incidence rate. Several factors may explain the increased rate of healthcare-related infections in the population of ICU patients with COVID-19, including structural factors such as the introduction of new ICU beds, organizational factors such as the inclusion of new teams of physicians and nurses without prior intensive care experience, and functional factors like changes in patient care standards [21]. All of these structural, organizational, and functional changes were present in the dedicated ICU ward for COVID-19 that we investigated.

One of the most common clinical forms of infections in Polish ICUs is nosocomial pneumonia (NP) [22, 23]. In studies conducted before the COVID-19 pandemic in southern Poland, the frequency of nosocomial pneumonia ranged from 4 to 10% [8, 9], [24, 25]. However, a considerably higher incidence rate (17%) of hospital-acquired pneumonia was reported in the period before the COVID-19 pandemic (2017–2018) by Dubiel et al. [23] in a study that involved 11 Polish ICU wards located in the northern region of Poland. According to the ECDC report [26] from studies conducted before the COVID-19 pandemic in European countries from 2008 to 2012, the average incidence rate of NP in ICUs was 6%. Other ECDC reports [10, 22] from studies conducted in European ICUs also indicated a 6% incidence rate of NP.

During the COVID-19 pandemic, significantly higher incidence rates of nosocomial pneumonia were reported among patients hospitalized in the ICU due to COVID-19. In our study, the incidence rate of NP in this group of patients was 14% and it was almost twofold higher compared to the group of non-COVID-19 patients (8%). Kozłowski et al. [18], in their study involving two ICU wards in northern Poland, reported a frequency of nosocomial pneumonia in patients treated for COVID-19 as 30%. Conversely, in a single-center ICU study conducted by Bardi et al. [20] in Spain, the incidence rate of nosocomial pneumonia in COVID-19 patients was 23%.

In our study, we calculated the VAP incidence rate per 100 patients treated in the ICU, which was 8% for both non-COVID-19 and COVID-19 patients. The VAP incidence rate obtained in our study aligns with results from other studies. Chinese studies from Wuhan reported a VAP incidence rate per 100 patients treated in the ICU due to COVID-19 at 31% [27]. Italian researchers observed an even higher VAP incidence rate of 50% [18]. A systematic review and meta-analysis conducted by Ippolito et al. [28] estimated the overall VAP frequency in patients treated in the ICU due to COVID-19 to be 45%. The elevated incidence of hospital-acquired ICU infections among patients with COVID-19 may be attributed to their increased susceptibility to lung tissue infections by bacteria present in the ICU environment, owing to the initial damage caused by SARS-CoV-2 [29]. Patients admitted to the ICU often had acute pneumonia due to SARS-CoV-2, accompanied by respiratory distress syndrome, comorbidities, and advanced age [20]. In many cases, a majority of patients (96%) [20], and even the entire cohort in some investigations [18], required invasive mechanical ventilation, a significant risk factor for VAP. It has been demonstrated that intubation and mechanical ventilation can increase the risk of pneumonia by 6 to 21 times [30].

In our study, 292 (70%) of the patients required mechanical ventilation of lungs. Interestingly, we observed a higher incidence of hospital-acquired pneumonia related to ventilation (VAP) in the group of COVID-19 patients, with an incidence rate density of 14/1000 days of ventilation, compared to the group of non-COVID-19 patients where it was 4/1000 days of ventilation. This increased VAP incidence rate in the COVID-19 patient group aligns with findings from Maes et al. in Cambridge, Great Britain [31], who reported rates of 28/1000 days of ventilation for COVID-19 patients compared to 13/1000 days of ventilation for non-COVID-19 patients.

COVID-19 patients frequently require prolonged invasive mechanical ventilation (MV), involving prone positioning, heavy sedation, and muscle blockers for several weeks. Furthermore, there is substantial evidence of prolonged immunosuppression, including deep lymphopenia [32]. This accounts for a high risk of secondary hospital-acquired infections, primarily ventilator-associated pneumonia (VAP) [33]. Diagnosing ventilator-associated infections remains a challenge, primarily due to the significant heterogeneity in clinical presentations. There is currently no consensus on appropriate diagnostic strategies for VAP. Regardless of the definition, a precise diagnosis of VAP necessitates clinical signs of infection, microbiological evidence, and chest X-ray findings. However, the interpretation of the latter can be complicated by pre-existing parenchymal injuries [34].

In our study, bronchoscopy was performed in only 5% of COVID-19 patients and 20% of non-COVID-19 patients. The basis for microbiological VAP diagnosis in COVID-19 patients was derived from material obtained from the lower airways in all 18 cases, using a diagnostic approach known as non-protected sample with quantitative culture (PN2). A study conducted before the COVID-19 pandemic, involving seven Polish ICU wards, observed that the duration of treatment for VAP patients who were correctly diagnosed using PN1 was shorter [34]. There was also a notable shift over time in the microbiological diagnostic methods employed for VAP patients. Notably, *A. baumannii* was predominantly observed in VAP cases diagnosed using substandard methods (non-PN1) [35]. The clinical presentation of COVID-19 pneumonia tends to be relatively uniform, commonly featuring high fever, hyperleukocytosis, severe hypoxemia, extensive bilateral radiologic infiltrates, and biological inflammatory syndrome. Given the similarity in presentation between COVID-19 pneumonia and VAP, the traditional diagnostic criteria for VAP are not applicable to the critically ill COVID-19 population [33]. Performing fiberoptic bronchoalveolar lavage in severely hypoxemic COVID-19 patients is often impractical due to the inherent risk of exacerbating hypoxemia. As a result, many ICUs resort to less invasive endotracheal aspirate (ETA) sampling with quantitative or semi-quantitative cultures, even though these methods may be less reliable for determining the necessity of antibiotic treatment. It is exceedingly challenging to distinguish between COVID-19-associated ARDS with asymptomatic bacterial colonization and a true VAP based solely on traditional threshold values, such as the 10^5^ CFU/ml for ETA samples [33]. These microbiological diagnostic challenges contribute to distinct differences in VAP classification and diagnosis in patients with COVID-19.

The precise identification of COVID-19 patients in need of new antibiotics for clinically relevant bacterial superinfections is a challenging task, which often results in the overuse of broad-spectrum antibiotics, even in the absence of supporting data in the literature [36]. Consequently, the majority of ventilated COVID-19 patients with ARDS receive prophylactic antibiotics as a preventive measure against undocumented VAP. This strategy carries a substantial risk of selecting multi-drug-resistant bacteria or even fungi, particularly in patients expected to remain on invasive MV for a long period [33].

The predominant causative agent of infections in our study was *Acinetobacter baumannii*, accounting for 36% of cases. However, in the group of patients with COVID-19, this microorganism was responsible for 63% of infections, whereas in the non-COVID-19 group, it accounted for 24% of infections. Previous Polish studies have consistently reported the frequent isolation of *Acinetobacter baumannii* in ICUs [9, 25, 37]. In a study by Kozłowski et al. [17], *Klebsiella pneumoniae* and *Acinetobacter baumannii* were identified as the most common pathogens responsible for VAP. Another study conducted by seven Polish ICUs from 2013 to 2015 found that *Acinetobacter baumannii* was primarily associated with VAP cases diagnosed using suboptimal methods (non-PN1) [35]. The concerning observation in our study is the increasing trend in the incidence rate of *Acinetobacter baumannii*. In 2020, it accounted for 10% of cases, rising to 13% in 2021 (OR = 3.342, 95% CI 1.799–6.208, *p* < 0.001). It is noteworthy that the incidence rate of *Acinetobacter baumannii* in patients admitted to our investigated ICU between 2012 and 2019 was 4%. An important characteristic of *Acinetobacter baumannii* is its ability to survive in dry conditions for extended periods, making the hospital environment a significant reservoir for this microorganism. It is suggested that *Acinetobacter* is more likely to cause infections in facilities with older infrastructure [23].

In our study, the mortality rate among COVID-19 patients was 64%, which was more than four times higher compared to non-COVID-19 patients (16%). Furthermore, significant disparities in mortality were noted among patients with HAI: in the COVID-19 group, a nearly twofold higher mortality rate of 21% was observed compared to 12% in the non-COVID-19 group. This pattern aligns with the findings of Kozłowski et al. [18], who reported a 72% mortality rate in COVID-19 patients with HAI versus 65% in those without HAI. Notably, a multicenter Italian study reported a 30% mortality rate among COVID-19 patients [19]. Bardi et al. [20] reported a 36% mortality rate in a university clinic in Madrid and highlighted a significant association between HAI and patient mortality. Specifically, the death rate was 54% in the group of patients with HAI compared to 24% in the group without HAI.

Hospital-acquired infections are a common complication in patients with COVID-19 treated in the ICU, which may contribute to the elevated mortality observed in this patient population [20].

In our study, it was also observed that among patients with NP, the mortality rate in the group of COVID-19 patients was almost twice as high compared to the non-COVID-19 group, at 10% versus 4%, respectively. This pattern is consistent with the findings of Maes et al. [31], where the mortality rate in the COVID-19 patient group with VAP was nearly twice as high as in non-COVID-19 patients, with rates of 38% versus 21%. According to a meta-analysis of 20 studies, the average mortality rate due to VAP in the group of COVID-19 patients was 43% [28]. It appears that critically ill COVID-19 patients, hospitalized in the ICU, grappling with acute viral infections, often necessitating mechanical ventilation and other invasive treatments, and exposed to multidrug-resistant strains that colonize the ICU, frequently face a challenging battle for survival.

### Limitations of the study

Our study has several limitations. The most significant of them include its single-setting nature, the relatively small sample size and the short-term duration of the study. Another notable limitation is the absence of data on comorbidities.

## Conclusions

In patients treated in the ICU with COVID-19, the incidence of PN and VAP and the risk of *Acinetobacter baumannii* infection were much higher than in patients treated in the ICU for reasons other than COVID-19. Although high, the risk of infections in our study was similar to the results reported by other authors. However, the proportion of *Acinetobacter baumannii* correlated with sub-optimal sample type for microbiological diagnostics. This observation indicates important challenge for infection control which is improving microbiological diagnostics methods and cooperation with infection control team and microbiological laboratory.

## Data Availability

The datasets used and/or analyzed during the current study are available from the corresponding author upon reasonable request.

## Abbreviations

SARS-CoV-2: Severe Acute Respiratory Syndrome Coronavirus 2
HAI: Healthcare-Associated Infections
ICUs: Intensive Care Units
PN: Hospital-acquired pneumonia
BSI: Hospital-acquired bloodstream infection
UTI: Urinary Tract Infections
SSI: Surgical Site Infections
GI: Gastrointestinal Infections
SST: Skin and Soft Tissue Infections
LRI: Lower Respiratory Tract Infections
SYS: Systemic Infections
ARDS: Acute Respiratory Distress Syndrome
MV: Mmechanical ventilation
VAP: Ventilator-Associated Pneumonia
ECDC: European Centre for Disease Prevention and Control
